# Socio-Ecological Barriers to Dry Grain Pulse Consumption among Low-Income Women: A Mixed Methods Approach

**DOI:** 10.3390/nu10081108

**Published:** 2018-08-17

**Authors:** Shelly M. Palmer, Donna M. Winham, Ann M. Oberhauser, Ruth E. Litchfield

**Affiliations:** 1 Food Science & Human Nutrition, Iowa State University, Ames, IA 50010, USA; spalmer@iastate.edu (S.M.P.); litch@iastate.edu (R.E.L.); 2 Sociology, Iowa State University, Ames, IA 50010, USA; aober@iastate.edu

**Keywords:** legumes, beans, poverty, attitudes, cooking time, food security, nutrition education

## Abstract

The purpose of this study was to determine the socio-ecological influences on dry grain pulse consumption (beans, peas, lentils, chickpeas) among low-socioeconomic women in Iowa. Seven focus groups were conducted, with 36 women who qualified for income-based federal assistance. Data were collected from October 2017 to January 2018. Participants completed a survey that gathered individual demographics, assessed perceptions of dry grain pulses, and level of food security. Fifty-eight percent of the women were non-Hispanic white, and 39% were African American, all with an average age of 34.7 years. Thirty-three percent of the women consumed pulses less than once per week. Over 80% agreed that beans were healthful and satiating. Some health benefits of beans were unknown by more than 33% of the population, e.g., lower cancer risk, lower LDL, maintain blood glucose. Only 30% of the women were food secure. Focus group audio recordings were transcribed and analyzed by two researchers, using the grounded theory approach. At the policy level, participants knew pulses were included in USA federal nutrition assistance programs like the Special Supplemental Nutrition Program for Women, Infants, and Children (WIC). Pulses were widely available in grocery stores in communities. Interpersonally, women felt that male partners preferred meats, and children needed animal-source proteins. Individually, women perceived uncooked dry pulses were challenging to prepare. Conclusively, more detailed instruction on pulse preparation, different pulse varieties, and offering canned pulses through WIC may increase consumption.

## 1. Introduction

Living below the federal poverty level poses an increased risk of many chronic disease conditions, including heart disease, stroke, cancer, and type 2 diabetes. Multiple factors influence an individual’s risk of chronic disease including lifestyle, nutrition, food availability, education level, socioeconomic status, ethnicity/race, and genetic predisposition [1]. Women commonly make food choices in households based on family taste preferences, time restrictions, convenience, familiarity, and culture, rather than the nutrient composition of foods alone [2]. In addition to lifestyle and health behaviors, socio-economic status (SES) influences an individual’s risk for chronic disease.

SES encompasses an individual’s financial well-being, education level, and employment status. Studies suggest low SES can significantly affect an individual’s quality of life and may decrease life expectancy by up to 8.5 years [3]. While theoretically modifiable, educational level of an individual regulates access to occupations, ability to use resources, household income, and social status. Additionally, employed individuals may feel more secure about the future and have less physical deterioration with age [4]. However, economic well-being, attaining higher education, and gainful employment can be difficult to obtain for individuals who are living in poverty [5]. Economic hardships often create stress around basic needs such as food, shelter, clothing, and care which can in turn lead to or exacerbate health problems [3].

**Figure 1:**
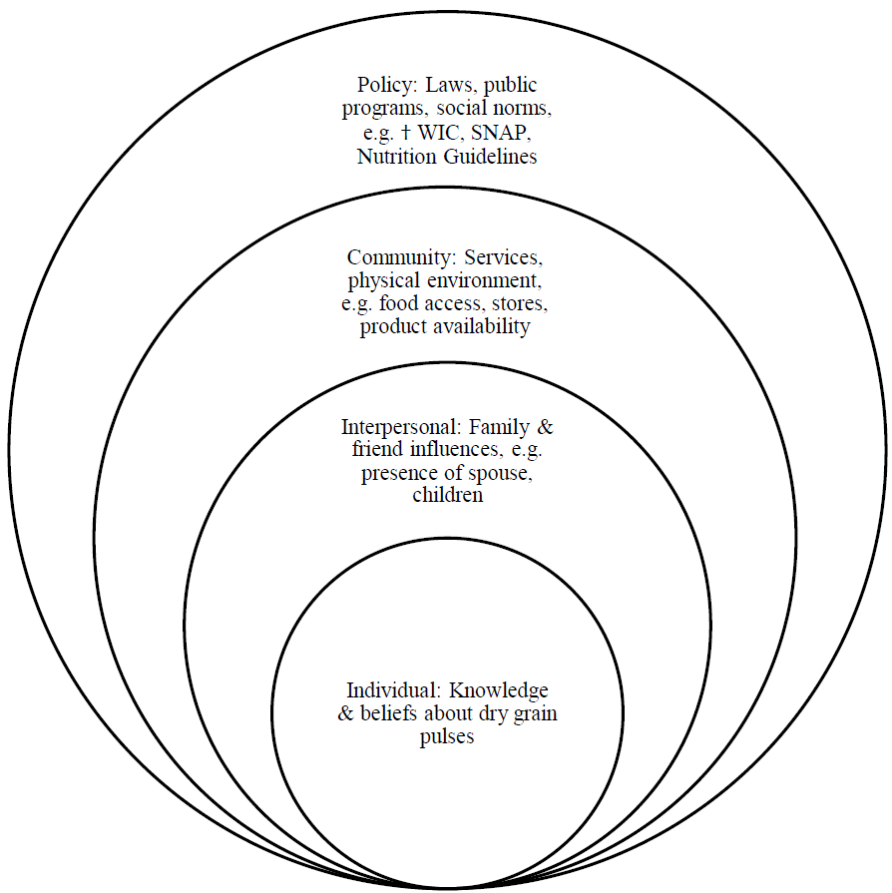
Levels of influence along the socio-ecological model used to determine women’s perceptions of dry grain pulses (adapted from Bronfenbrenner and McElroy) [6,7]. † WIC = Special Supplemental Nutrition Program from Women, Infants, and Children; SNAP = Supplemental Nutrition Assistance Program.

SES extends far beyond personal health behaviors, and includes external and structural influences on an individual, such as their family and environment. The socio-ecological model assesses individual, interpersonal, community, and policy levels of influence [6,7]. The inner-most level includes the individual’s knowledge, beliefs, and interactions with their immediate environment. The interpersonal level includes social influences with whom the individual interacts with regularly, such as family, friends, and social networks. The third layer is the community level including the structure, support, and physical environment provided by resources where individuals live. The outer-most level are the policy influences, such as laws, regulations, public programs, and social norms set forth that guide individual behaviors [6,7] (Figure 1). The socio-ecological model provides an appropriate framework for assessing food consumption in terms of policy, food availability, and familial influences, and individual choices. In this study, the model framework is used to determine influences at each level regarding dry grain pulse consumption.

Dry grain pulses are harvested as seeds and frequently consumed as staple grains [8]. This food group includes beans (pinto, black, navy, kidney, white), peas, lentils, black eyed peas, and chickpeas (garbanzos) among others. Most pulse varieties are high in fiber, protein, folate, iron, zinc, potassium, and magnesium. Documented health benefits of some market classes include reduction of low-density lipoproteins and total cholesterol, lowering of postprandial glucose, increased satiety, and possibly lower cancer risk [9,10,11,12,13]. Noting the nutritional and health benefit aspects of pulse consumption, the Dietary Guidelines for Americans (DGA) have recommended increased pulse consumption since 2005. The current DGA recommendation for women aged 19–50 is 1.5–2 cups of pulses per week, which is typically not met. Recent national intake estimates are only 0.5–1 cup per week [14]. If pulse consumption was increased among low SES women and their families, dietary quality could be improved by increasing intakes of several shortfall nutrients [8,9]. In the U.S. Midwest only 13% of the population consumes any dry grain pulses, with Hispanics accounting for the majority of regional intakes [10].

Although national survey data indicates pulse consumption is low, little evidence exists about the barriers and motivators to pulse consumption among low SES women. A few studies have focused specifically on beans, but not the broader group of pulses. A survey of 136 mostly white WIC recipients in Florida showed women did not think of beans as being a replacement for meat products, but rather as an addition [15]. Among 228 predominately Hispanic low-income women surveyed in Arizona, the majority did not know of bean health benefits such as lowering cholesterol, cancer risk, and postprandial blood glucose [16]. A recent Iowa survey with 158 low SES Hispanic and non-Hispanic white women showed similar findings as in Arizona with limited knowledge of health benefits [17]. In all three surveys, the majority of respondents agreed that beans help improve nutrition and promote satiety indicating positive perceptions of pulses [15,16,17].

While previous survey research on beans describes in part what low SES women think about them, qualitative studies allow for in-depth breadth of observation and hypothesis generation. Focus group discussions can give greater understanding to the motivators and barriers for a phenomenon like low pulse consumption as the method allows for extensive analysis as a representational approach [18]. No published qualitative studies assessing why low SES women, or other groups, do not consume beans or pulses were found. Understanding the influences of food purchasing from the perspective of low SES women within their households, community, and greater policy environment can inform researchers on testing the appropriateness and effectiveness of nutrition education interventions. This type of intervention is an effective way to change health behaviors because it resonates with the audience and is grounded in the reality of daily experiences [19]. In sum, the current study investigates elements of the socio-ecological model in order to identify barriers to and motivators for pulse consumption. Low SES women of reproductive age were chosen due to their potential influence in shaping food preferences in households, the development of taste preferences in children, and nutrition support for parents [20].

## 2. Materials and Methods

### 2.1. Survey Instruments

Participants completed questionnaires on demographics, bean perceptions, and food security status. Demographic questions were drawn from three sources and included Hispanic ethnicity, race, and household composition from the Expanded Food and Nutrition Education Program (EFNEP), [21] education and marital status from the American Heart Association Women’s Survey, [22] and employment status from the Behavioral Risk Factor Surveillance Survey (BRFSS) [23]. Urban or rural classification of residence was based on participants’ assessment of their location. Food security was measured according to the six-item USDA core food security module [24]. The bean consumption frequency wording was from a food frequency screener for fruits, vegetables, and fiber [25]. The nine bean health benefit questions were used from surveys administered to a similar demographic in Arizona and Iowa [16,17]. These questions utilized a Likert-type scale (strongly disagree, disagree, agree, strongly agree, and do not know option).

### 2.2. Focus Group Design

A total of seven focus group discussions with 36 low SES women in central Iowa were conducted. An interview guide was followed which was developed based on a literature review, and findings from previous survey research on knowledge, attitudes, and perceptions of beans with similar groups of low SES women in Iowa and Arizona [16,17,26]. Using the socio-ecological model, the interview guide was based on five themes: Individual consumption patterns, individual knowledge of beans (pulses), social consumption patterns, physical environment (food access), and policies (Table 1) [6,7]. As an ‘ice breaker’ at the beginning of each focus group, the moderator defined pulses, and showed real canned and bagged varieties of pulses. These were left on display during the focus group discussion. The next group exercise was a meal demonstration using food models and the five food groups from MyPlate (dairy, protein, grain, vegetable, fruit) [14]. One demonstration meal had chicken and in the second example, kidney beans were substituted for the chicken. Participants were asked to explain their perceptions of suitability, and attitudes about the two meals. The interview questions and activities were pilot tested with a group of seven Expanded Food and Nutrition Education Program (EFNEP) educators. Minor modifications were made for clearer interpretation and flow before official data collection.

### 2.3. Participant Recruitment

The Iowa State University Institutional Review Board approved the study procedures. Recruitment flyers were displayed in health care clinics, WIC offices, food pantries, libraries, Extension program sites, and other community resource agencies where services are provided to low SES clients. Potential participants expressing interest were screened to ensure they were females between the ages of 18–50 who primarily speak English, and received income-based assistance. Screening information was sorted by preferred locations, dates and times available to generate focus groups of 5–10 women, and possible alternates. The day before the session, participants were sent a reminder email and contacted by telephone to confirm their attendance, location, and time. If someone cancelled, an attempt was made to find a replacement from the alternate list.

Upon arrival at the focus group, women received a printed copy of the informed consent. A researcher read the consent form aloud to the group and asked if there were any questions prior to signing. Survey instruments were completed prior to the focus group session. Researchers had no previous relationships with the participants. Investigators were trained according to the Krueger methodology and three practice focus groups were conducted prior to data collection [18,27]. All focus groups were moderated by the lead author (SMP). Two other trained researchers assisted with the sessions and took detailed field notes. All participants received $40 cash as an incentive at the end of the focus group. Upon the seventh focus group, discussion topics were being repeated signaling to the research staff, that saturation had been reached.

### 2.4. Qualitative Analysis

Audio recordings of the focus groups were transcribed and uploaded into NVivo version 11.0 (QSR International Pty Ltd., Doncaster, Victoria, Australia) to aid in qualitative analysis. The moderator and one field note researcher independently read the transcripts and collaborated to develop a codebook. The final codebook reflected the socio-ecological model, interview guide, and themes originating from the discussion content. These themes included: Individual pulse consumption, pulse consumption by family or friends, nutrition information, and policy issues. The grounded theory approach to qualitative analysis was applied to define themes [28]. There were no unresolved disagreements in coding as the two reviewers collaborated to develop and refine themes after initial coding. Inter-rater reliability between the two coders was high, 99.59, with a kappa value of 0.83 [29].

### 2.5. Quantitative Analysis

Survey data were entered into SPSS Version 24.0 (IBM Corp., Armonk, NY, USA). Frequency distributions of variables were examined for normality. Bivariate statistics using Chi-square or ANOVA were compared by race (White or African American/Other) and food secure/food insecure. The nine items from the bean health benefits statements were examined using principal components analysis with varimax rotation. The scree plot indicated one underlying construct representing participant’s knowledge on the bean health benefits with an eigenvalue of 4.15 and explaining 69.1% of variance [30]. The six items from the rotated factor plot had a resulting Cronbach’s alpha of 0.90 indicating high reliability. Self-reported heights and weights were used to calculate BMIs which were categorized into underweight, normal, overweight, and obese based on current classifications [31]. Responses from the USDA food security module were totaled and categorized as high food security, low food security, and very low food security [24].

## 3. Results

### 3.1. Quantitative Results

Table 2 includes demographic characteristics of the 36 women that participated in seven focus groups. Fifty-three percent of the participants identified as white (*n* = 21), and 39% (*n* = 14) as African American. One women identified as Asian, and two white women self-identified as Hispanic. The food assistance program used by the greatest number of participants was the Supplemental Nutrition Assistance Program (SNAP), (47%), followed by WIC (36%), and child nutrition programs (31%) such as reduced-price breakfast and lunch. Ten of the 36 women (28%) participated in more than one food assistance program. Almost 70% of the women were food insecure and reported an annual household income of $24,999 or less. There were no significant differences in food security by race, education, marital status, employment, or income.

Table 3 shows frequency of bean consumption and knowledge on bean health benefits and statements of bean knowledge among participants. Only 17% of the women reported consuming pulses 4–6 times per week or more, or the recommended amount according to the DGA. Thirty-six percent of the participants ate pulses 2–3 times per week, while the majority (47%) ate them once per week or less, if at all. African American women reported higher pulse consumption than White women. Over 80% agreed or strongly agreed that eating beans improves your nutrition, helps you feel full, and can give one gas. Many women did not know that bean consumption could decrease some cancer risks (56%), help control blood sugar (39%), or lower ‘bad’ cholesterol (33%). Eighty-one percent agreed eating beans can cause intestinal gas. Significant differences were found between frequency of bean consumption and responses to statements that beans contributed to weight loss, lowering cholesterol, healthy GI system, and being good for people with diabetes. The ‘knowledge of health benefits’ scale was normally distributed with a mean of 13.8 ± 7.1 (range 0–24). Women reporting higher bean consumption had greater knowledge of bean health benefits (*p* = 0.044).

### 3.2. Qualitative Results

#### 3.2.1. Individual

Participants’ knowledge of pulses and consumption patterns were discussed from a variety of perspectives. For example, when shown examples of canned black beans and garbanzo beans, dry lentils and green split peas, participants were most familiar with black beans. Some participants did not know garbanzo beans and chickpeas were synonymous, or how these pulses are used. In general, knowledge of lentils and split peas was very limited.

Knowledge about preparation methods of dry pulses was lacking unless the individual grew up rehydrating and preparing them. One major barrier to utilizing dry pulses was the amount of time it takes to soak, cook, and prepare a dish using them. One participant explained how she grew up cooking dishes with dry pulses, and when they were in a hurry they purchase a can instead.

“*I don’t really know how to use the ones in the bag. Like my mom always used the ones in the can... that’s how I know how to cook with them.*”

[age 31, rural]

“*No one has ever taught me, if somebody did, then it would be a different story but I don’t know what it’s supposed to be like and when it’s right and when it’s not.*”

[age 35, urban]

“*I would never like spending my time trying to cook them. Unless someone is bored the night before and they put them in the crockpot.*”

[age 39, rural]

Participants receiving WIC benefits were more likely to have tried preparing dry pulses. After a bad experience, such as not letting them soak long enough, undercooking, a hard texture after cooking, or family members expressing dislike, they may be less likely to try them again.

“*I have to use the pressure cooker, and I’ve had two bad incidents with it so I just stopped cooking beans for a while.*”

[age 35, rural]

One of the most common pulse varieties consumed by participants was canned pork and beans. Participants noted they are convenient, satiating, inexpensive, and taste good with the sauce. The most common dishes made with pulses were soups and dips, rather than as a side on their plate. Women also expressed interest in having canned beans available through their Iowa WIC benefits.

Participants knew pulses were a good source of protein and fiber. In two of the seven discussions, women reported personal stories of pulses being a good source of iron, and through the addition of pulses in their personal diets were able to raise blood iron levels.

“*I was vegetarian for 2 years, so I replaced a lot of my meat with beans and before I always tried to donate blood but my iron was always too low, but after being vegetarian for 2 years, I was able to donate blood because my iron was perfect. I have to think it was because of the beans.*”

[age 48, rural]

#### 3.2.2. Interpersonal

In general, women’s time was limited with work responsibilities, meal preparation, doctor’s appointments, and often relying on federal assistance for food and healthcare. These factors contributed to the need for quick preparation of food.

“*I’ve got three little babies, and I mean you want something that’s quick, easy, fast because I’ve got an hour and a half from when I get home and put my kids to bed... it’s frozen pizza, fish sticks, chicken nuggets, crap like that.*”

[age 29, urban]

Women noted their household composition had a major influence on the foods they purchase. Participants reported consuming meat because their husband or children wanted meat as the main dish of the meal. Despite cases where the husband preferred meat at every meal, meat was not always accessible or was considered too expensive to purchase, especially at the end of the month. Participants, such as one woman quoted below, reported buying beans only at the end of the month because they were cost efficient to feed their families as well as satiating for their children. Several woman voiced concerns about the stigma of beans as associated with poverty, or as a food used as the last resort to hunger.

“*...his philosophy is you plan the meal around the meat, not the other way around. I ate vegetarian for a couple of years, so I would eat that but he wouldn’t, he would need a piece of meat.*”

[age 48, rural]

“*I would use beans as a poor meal at the end of the month. If I don’t have any money, I’ll get those smoked sausage rings... and cook them with the beans and rice and my children go to sleep pretty good too.*”

[age 29, urban]

“*People give beans a lot of bad rap, oh like that’s the poor man’s food, oh they are nasty…*”

[age 39, rural]

During the full-size meal demonstration, participants indicated the chicken-based meal was most similar to what they would feed their families, making sure the chicken was baked not fried and stating a greater preference for white rice than brown. When the chicken was replaced with kidney beans, participants suggested it was “light” and would not be satiating. The most frequent solution to making the demonstrated meal more like what the women would serve was to add the chicken to the meal and mix everything together (chicken, rice, beans, peas) and add some spice.

#### 3.2.3. Community

According to the participants, grocery stores are the most common place to shop for beans. In each of the focus groups, participants said beans were available in the grocery or convenience stores where they regularly shop. Dry pulses were most frequently found in the ethnic foods aisle, whereas canned beans were near the canned vegetables, or the rice and pastas. Supermarkets were the most frequently mentioned place to buy groceries, because they can go to one place and purchase household needs and food. Discount grocery stores were mentioned in six of the focus groups as offering food at a reduced price. The major determinants for choosing which grocery store to shop at were: Family influences, neighborhood location of grocery store (safety), price of food, convenience (proximity) of grocery stores, sales and advertisements for food products, and nutritional composition of products sold at a store.

“*I will go to four grocery stores in a week if it saves me money. I am not loyal to any grocery store.*” [age 21, rural].

#### 3.2.4. Policy

Women used SNAP benefits to purchase beans and stated it was simple as long as the funds were there to support it. Those receiving SNAP noted funds do not last the whole month, and either their family waits until the funds are renewed, visits food distribution centers, or uses funds not used for other expenses to buy food. WIC food purchasing habits were discussed in four of the seven focus groups. Legumes in 1-pound bags are eligible using WIC benefits. Here again, participants voiced concerns that they did not know how to cook them.

“*You buy the white bread instead of the wheat bread because it saves you the 12 cents a loaf... the 12 cents adds up.*”

[age 21, rural]

“*...I have known several families that have been on WIC and when they get those checks typically they don’t use the bean one... because they don’t know what to do with them, or they just don’t eat them.*”

[age 48, rural]

Participants had a difficult time identifying any nutrition guidelines that encourage pulse consumption. Women were from a variety of educational backgrounds and were not asked about prior nutrition education. At least one woman in every group was aware of the MyPlate graphic. Discussants reported seeing the graphic in grocery stores, WIC clinics, and Extension programming. The focus group moderator asked the women about MyPlate, and there was a general idea of each of the five food groups. In all seven focus groups, the women knew that beans fit in the protein group, but some said they belong in the grains group, and a few stated they belong in the vegetable group.

## 4. Discussion

The objective of this study was to examine barriers and motivators to bean consumption among low SES women using the socio-ecological model construct [6,7]. Results suggest major knowledge gaps in previous nutrition education regarding pulse consumption. Some current messaging promotes beans as a low-cost replacement to animal-source proteins, which may actually dissuade consumers from purchasing them. Focus groups revealed that although low SES women are conscious of food costs, they do not want the food they buy for their families to be perceived as ‘cheap.’ Furthermore, women did not regard beans as a protein replacement for meat sources. These findings can inform the development of tailored nutrition education programming for this audience.

Previous research suggests interventions targeting cooking skills resulted in positive change among low SES participants [32,33,34]. In these studies, confidence of cooking skills increased as well as the number of vegetables prepared. Successful interventions for low SES individuals are shorter in duration and length of time to retain participant’s attention [31]. Overall, tailored nutritional messages have had a greater effect on increasing fruit and vegetable consumption among low SES populations than more general signs, pamphlets and written information [35]. The same may be true for dry grain pulses. Active messaging in nutrition education include: Hands-on demonstrations, recipe tasting, information on simple cooking methods, and rehydrating pulses.

Household nutrition is often compromised when consumers eat more food outside the home and can lead to diminished cooking skills [35]. Many people may consume greater amounts of convenience foods due to prevalence of fast food restaurants, savory taste profiles, lower cost, and time constraints for food preparation [5]. While the dietary pattern may not be unique to low SES groups, they have an increased risk of heart disease, diabetes, and total number of chronic diseases than others with high SES due to added stressors and limited resources [1,5].

Although participants in the current study knew beans were a good source of protein and fiber, there were knowledge gaps in terms of specific health benefits such as decreasing risk of high cholesterol, cancer, and moderating blood glucose in persons with type 2 diabetes. This was similar to findings in knowledge, attitudes, and perceptions of 158 Iowa women surveyed where almost 60% did not know bean consumption could lower LDL cholesterol, and 59% did not know about maintenance of blood glucose values [17]. When asked why beans should be purchased, the focus group respondents reported they were good for the digestive system and they were a suitable addition to meals at the end of the month because they were inexpensive and nutritious.

Results from this study revealed the participants had limited knowledge in cooking methods, and how to incorporate pulses into their everyday diets. Those women who did not know how to cook dry uncooked beans did not use their food assistance vouchers for them either. These findings contrast with survey responses from Florida WIC participants who expressed confidence in bean preparation and incorporation methods [15]. Further investigation of these perspectives with greater diversity of cultures, geography, and resources is needed.

Many of the women reported their spouse and children-influenced household grocery shopping practices. They were cautious in purchasing foods to limit food waste and keep their children full and not hungry at night. Beans were the most frequently known pulse. Women were less familiar with other varieties that can have faster preparation time from dry such as chickpeas, or lentils. Consumption of canned pork-and-beans was most common. These results suggest women, especially those in WIC, are interested in learning how to incorporate these sustainable sources of protein in their everyday diets. Thus, hands-on taste testing at community sites or as part of nutrition education through Extension or WIC could improve knowledge and increase exposure to other pulses like lentils. A pilot program that combined taste testing with hands-on preparation curriculum like “Spillin’ the Beans” developed by Garden-Robinson and Whigham showed success in increasing bean consumption among participating children and parents [34].

The versatility of beans in a wide variety of dishes is a motivator to increase the nutritional value of the dish. Serving suggestions such as adding to soups, casseroles, or other hot dishes could aid in improving dietary quality and reducing shortfall nutrients across the family unit. Beans were also widely available, and participants stated that any grocery store or food pantry they frequented provided access to beans, but they were unsure about lentils, peas, or chickpeas.

The qualitative results from this study suggest that nutrition education programs for low SES women may not be adequately addressing how to best utilize dry grain pulses. In a national convenience survey of 733 nutrition educators mostly from Extension, respondents had moderate knowledge of dry edible bean health benefits [36]. They reported their health knowledge, bean consumption level, and awareness of different bean types was higher than the clients they served. Among 296 Registered Dietitians (RD) in Arizona the majority knew most health benefits from bean consumption associated with chronic diseases. About 23% of the Arizona RDs said they ate beans three or more times per week which was similar to the 20% reported for the national nutrition educator sample. Neither survey specifically asked about other pulses beyond beans. Over 67% of the Arizona RDs did not know the definition of pulses, with almost a third unable to define the term legumes [37]. These survey findings indicate a possible disconnect among Extension nutrition educators and RDs on knowledge of different pulses and ability to promote them to clients. From a broader perspective, evidence that EFNEP and other nutrition education programs work to reduce food insecurity is strong [38].

To complicate matters further for low SES women in Iowa, the current WIC policy is to allow vouchers only for dry pulses, not canned. Following Institute of Medicine recommendations, the national WIC program added canned legumes to its revised food packages in 2006 to boost fiber, folate, and iron intakes in women and children, citing: “Allowing canned beans substitution accommodates participants’ preference for easier to prepare item and may encourage the consumption of beans” [39]. State WIC offices had leeway in adopting these guidelines with Iowa implementing them in 2009. Unfortunately, the Iowa WIC program stopped the canned option in 2012 due to cost concerns. Recent national WIC package changes have decreased the amount of pulses provided to allow for use of other more popular foods [40]. Current WIC redemption for the pulse voucher is 51%. It is not known if attempts to improve awareness or to facilitate utilization of pulses by WIC clients has been done by the agency overall.

This study contributes to the current literature using a mixed methods approach to describe low SES women’s perceptions on pulses. These results may influence the creation of nutrition education messages for low SES women on increasing pulse consumption. Due to the method of data collection, sample size and narrow geographic location, the generalizability of the findings and survey data are limited. These results represent urban and rural populations in Iowa, which are somewhat different than larger urban areas. Rural communities may experience additional hardships in terms of food availability and accessibility.

## 5. Conclusions

These mixed method results have identified barriers and motivators for pulse consumption as well as gaps in knowledge among low SES Iowa women. The findings will support WIC and Extension nutritional programming that encourage purchasing and consumption of economically viable, and sustainable sources of protein. Based on these results a tailored nutrition education program can be developed to address the health benefits and ways to use pulses among low-income families. These measures will work to improve diet quality and, and ultimately, health.

## Figures and Tables

**Table 1:** Sample focus group interview guide questions outlined by socio-ecological model socio-economic status (SEM) constructs.

Question	SEM Construct
Why do you purchase bagged or canned beans, peas, chickpeas, or lentils?	Individual
On a scale from 1–5, how much do you know about the nutrients in beans, peas, chickpeas, or lentils?	Individual
Explain your family’s attitude toward eating beans, peas, chickpeas, or lentils.	Interpersonal
Where do you/family shop for foods?	Community
Where are beans located in the stores you shop at?	Community
What are some nutrition guidelines that encourage bean consumption?	Policy
If someone wanted to buy beans and received supplemental food assistance, how is this done?	Policy

**Table 2:** Distribution of demographic and household characteristics of low SES Iowa women (mean ± SD, or percentage) (*N* = 36).

Characteristics	Total
Age in years	34.7 ± 8.8
Race	
African American	38.9
White	58.3
Asian	2.8
Household Location	
Urban	63.9
Rural	36.1
Marital Status	
Single	58.3
Married	32.5
Living with Partner	5.6
Divorced/Separated	11.1
Living Arrangements	
With parents	8.3
With spouse	33.3
With other family members	16.7
By yourself	36.1
With roommate in a dorm/ house/ apt.	5.6
Food Program Usage	
Child Nutrition	30.6
SNAP †	47.2
WIC †	36.1
Food Distribution	8.3
No Food Programs	11.4
Employment Status	
Employed	50.0
Homemaker	16.7
Student	8.3
Disabled and unable to work	13.9
Unemployed	11.1
Years of Education	
Did not graduate High School	11.1
High School graduate or passed exam	30.6
Some college credit	19.4
Associate degree	16.7
Bachelor’s degree	11.1
Master’s degree or higher	11.1
Number children under age 18	1.3 ± 1.5
Total Household Size	3.2 ± 2.0
Household Income per year	
Under $10,000	37.1
$10,000–24,999	31.4
$25,000–49,999	17.1
$50,000–74,999	14.3
Food Security	
High food security	30.6
Low food security	36.1
Very low food security	33.3

† SNAP = Supplemental Nutrition Assistance Program; WIC = Special Supplemental Nutrition Program from Women, Infants, and Children.

**Table 3:** Bean consumption frequency and knowledge of health benefits among low SES Iowa women (*N* = 36; %).

Consumption Frequency	Less than 1 Per Week	About Once Per Week	2–3 Times Per Week	4–6 Times Per Week	1+ Times Per Day
How often do you eat beans …?	33.3	13.9	36.1	13.9	2.8
**Eating beans can ….**	**Strongly Disagree**	**Disagree**	**Agree**	**Strongly Agree**	**Do Not Know**
1. Improve Nutrition	0	2.8	66.7	19.4	11.1
2. Help to Feel Full	2.8	0	63.9	19.4	13.9
3. Lower Bad Cholesterol	0	2.8	52.8	11.1	33.3
4. Control Blood Sugar	0	2.8	55.6	2.8	38.9
5. Healthy GI Tract	0	0	50.0	19.4	30.6
6. Good for Persons with Diabetes	2.8	2.8	47.2	13.9	33.3
7. Help Lose Weight	0	8.3	47.2	13.9	30.6
8. Produce Gas	0	11.1	52.8	27.8	8.3
9. Lower Cancer Risk	0	8.3	30.6	5.6	55.6
**Summary scale ^†^**		**µ ± SD**		**Range**	
Knowledge of bean health benefits (summary of items 1–6)		13.8 ± 7.1		0–24	

† The response category ‘Do not know’ was recoded as 0 for scale summation.
